# FBXO28 targets SNAI1 for ubiquitin-proteasomal degradation in non-small cell lung cancer

**DOI:** 10.1016/j.jbc.2026.113405

**Published:** 2026-08-13

**Authors:** Jingyu Lin, Xinran Qiao, Hua Liu, Yang Chen, Zhen Li, Xinyi Tong, Mi Shen, Rui Zhang, Ying Han, Jiajia Shen, Zhen Wang

**Affiliations:** 1 Department of Biochemistry, Institute of Medicinal Biotechnology, Chinese Academy of Medical Sciences & Peking Union Medical College, Beijing, China; 2 Department of Thoracic Surgery, Shanghai General Hospital, Shanghai Jiao Tong University School of Medicine, Shanghai, China

**Keywords:** FBXO28, SNAI1, NSCLC, UPS, EMT

## Abstract

SNAI1 is a central regulator of the epithelial–mesenchymal transition, which is highly unstable and degraded through the ubiquitin-proteasome system. Although a panel of E3 ubiquitin ligases responsible for SNAI1 degradation have been discovered, whether there exists other E3 ubiquitin ligase remains unclarified. In this study, we demonstrate that FBXO28 expression is downregulated in non-small cell lung cancer (NSCLC) tissues compared to adjacent non-cancerous tissues, correlating with beneficial patient prognosis. Specifically, FBXO28 physically interacts with SNAI1, promoting its polyubiquitination and degradation in a mechanism dependent on phosphorylation by glycogen synthase kinase-3*β* (GSK3*β*). Functional assays indicate that FBXO28 controls the half-life of SNAI1 protein, which are abrogated by proteasome inhibitor MG132 in NSCLC cells. Importantly, FBXO28 suppresses the migratory and invasive abilities of NSCLC cells, an effect that can be mitigated by GSK3*β* inhibition. We also present data to show that SNAI1, along with SNAI2, mediates the regulatory function of FBXO28 toward migration and invasion of NSCLC cells in mice and zebrafish xenograft models. Collectively, our findings elucidate a significant role for FBXO28 in regulating SNAI1 stability *via* the ubiquitin-proteasome system, thereby providing insights into therapeutic strategies aimed at constraining metastasis in NSCLC.

Non-small cell lung cancer (NSCLC) is a highly aggressive and lethal malignancy characterized by potent metastatic capacity and resistance to conventional therapies (1). Poorly defined regulatory mechanisms for NSCLC invasion and metastasis, compounded by a lack of effective early screening, present a major barrier to improving patient outcomes (2). Understanding the molecular mechanisms governing NSCLC progression and metastasis will facilitate the development of novel therapeutic approaches and help improve patient outcomes.

The epithelial–mesenchymal transition (EMT) is a critical biological process that enables tumor cells to dissociate from the primary tumor and disseminate to distant organs (3). As a central regulator of EMT, SNAI1 is well known to promote invasion and metastasis in NSCLC by activating EMT-associated gene expression programs, whose stability, transcriptional activity, and subcellular localization are tightly controlled by post-translational regulation (4, 5, 6, 7, 8, 9, 10). For example, several F-box proteins, including FBXL14, FBXO22, FBXO11, and FBXO31, which form the SKP1-CULLIN1-F-Box (SCF) complex to act as E3 ubiquitin ligases have been shown to promote SNAI1 degradation *via* the ubiquitin-proteasome system (UPS) in a panel of cancer cells (9, 10, 11, 12). However, considering SNAI1 is an extremely labile protein, whether there exists other F-box protein responsible for SNAI1 degradation remains to be defined.

As a member of the SCF family, FBXO28 has been demonstrated to play critical roles in the pathogenesis of chromosome 1q41q42 microdeletion syndrome, the maintenance of genomic stability and cell cycle, as well as in lipid metabolism and inflammation (13, 14, 15, 16). Moreover, FBXO28 exhibits either oncogenic or tumor-suppressive function in diverse tissue types of cancer *via* targeting a panel of substrates such as MYC, SMARCC2 and HIF-1*α* (17, 18, 19) and thus serves as a poor or beneficial prognostic biomarker in pancreatic, ovarian and gastric cancer (18, 20, 21).

In our recent work, we have identified FBXO28 as a critical inhibitor of EMT and metastasis in hepatocellular carcinoma (HCC) cells through targeting SNAI2, another pivotal EMT transcription factor, for proteasomal degradation (22). Notably, we simultaneously observed a reduced SNAI1 protein level upon ectopic expression of FBXO28 (22), prompting us to speculate that SNAI1 may serve as an unidentified substrate for FBXO28 as well.

Here, we show that FBXO28 recognizes GSK3*β*-phosphorylated SNAI1 and mediates its degradation *via* the UPS. We also present that SNAI1, along with SNAI2, mediates the regulatory function of FBXO28 towards migration and invasion in NSCLC.

## Results

### FBXO28 expression is downregulated in mesenchymal cancer cells and associated with beneficial prognosis in patients with NSCLC

**Figure 1:**
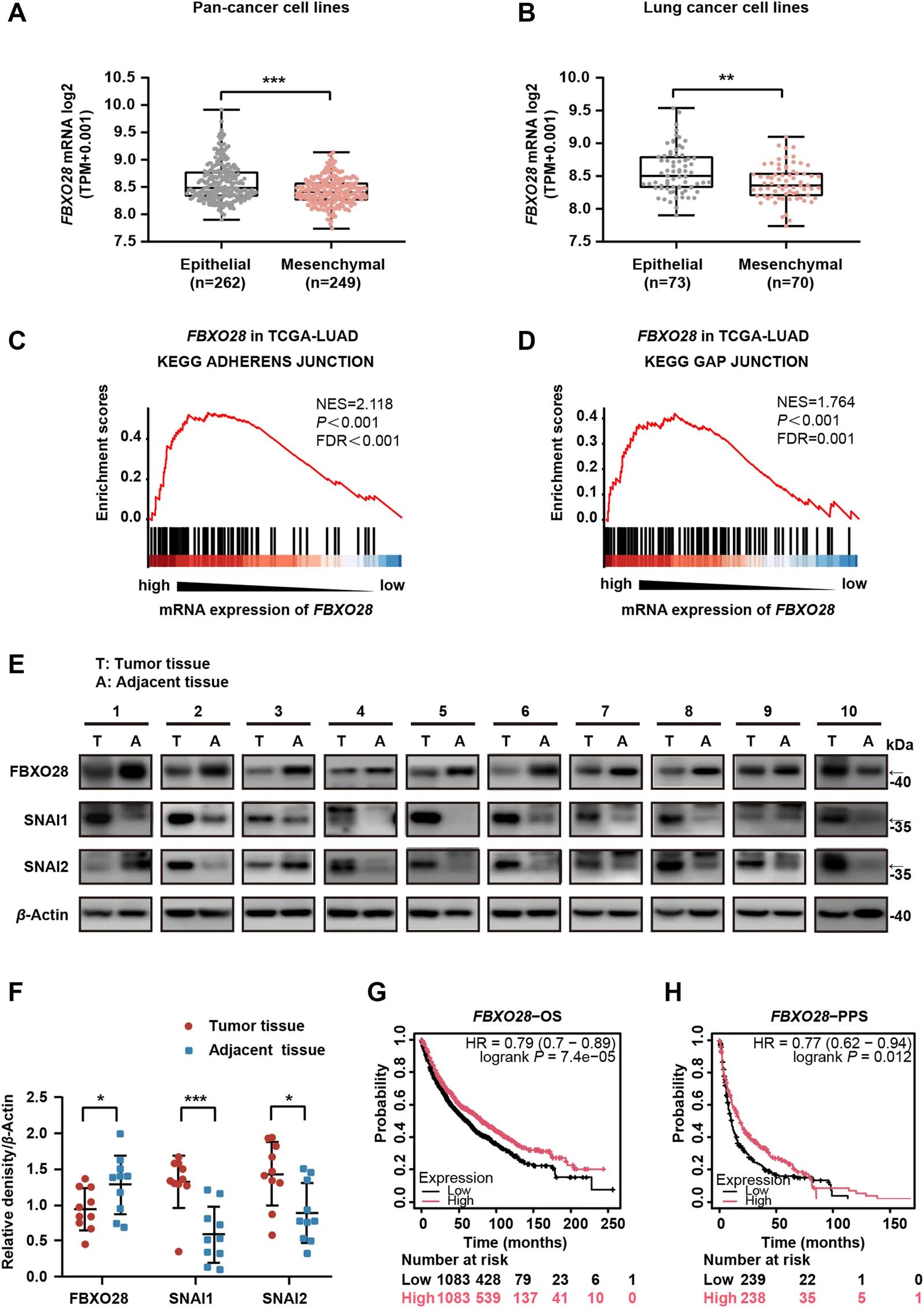
**Expression level of FBXO28 is negatively correlated with mesenchymal pan-cancer cells and lung cancer cells.***A*, *FBXO28* mRNA levels in epithelial and mesenchymal cell lines plotted based on the RPKM of human pan-cancer cell lines in CCLE. ∗∗∗*p* < 0.001 (independent *t* test). *B*, *FBXO28* levels in human lung cancer cell lines from CCLE. ∗∗*p* < 0.01 (independent *t* test). *C* and *D*, GSEA analysis of the TCGA datasets for Lung Adenocarcinoma (LUAD). *E* and *F*, protein levels of FBXO28, SNAI1 and SNAI2 in paired NSCLC specimens by IB analyses. The arrows indicated target bands. Quantification data (*F*) were expressed as mean ± SD (n = 10). ∗*p* < 0.05 and ∗∗∗*p* < 0.001 (paired *t* test). *G* and *H*, the Kaplan-Meier survival analysis of lung cancer patients retrieved from www.kmplot.com. Correlation of *FBXO28* expression with survival analysis in overall survival (OS) and post-progression survival (PPS) were shown in (*G*) and (*H*), respectively.

As our previous work focused on HCC (22), we first determined whether its regulatory function in EMT is universal in NSCLC. By classifying a panel of pan-cancer cell lines from the Cancer Cell Line Encyclopedia (CCLE) database into epithelial-like and mesenchymal-like subgroups, we found that *FBXO28* expression was significantly lower in mesenchymal-like cancer cells relative to that in epithelial-like cancer cell lines (Fig. 1*A*). A similar trend for *FBXO28* level in stratified subgroup of lung cancer cell lines was also observed (Fig. 1*B*). Moreover, gene set enrichment analysis (GSEA) from The Cancer Genome Atlas (TCGA) database NSCLC cohorts demonstrated that the gene expression levels of *FBXO28* positively correlate with the enrichment of adherens junction and gap junction signaling pathways (Figs. 1, *C*, *D* and S1, *A*, *B*). These data support a possibly negative regulation of FBXO28 in the EMT process of lung cancer.

Through Western blot analysis of 10 paired NSCLC clinical specimens, we found that FBXO28 protein levels were markedly lower in the NSCLC tissues relative to those in adjacent tissues, whereas SNAI1 and SNAI2 levels exhibited opposite trends (Fig. 1, *E* and *F*). These data support a broader role for FBXO28 in EMT regulation across multiple cancer types. Kaplan–Meier survival analyses revealed that lung cancer patients with higher *FBXO28* expression levels exhibited significantly prolonged overall survival (OS) and post-progression survival (PPS) (Fig. 1, *G* and *H*), confirming that *FBXO28* expression level is associated with beneficial prognosis in patients with NSCLC.

### FBXO28 interacts with SNAI1 and promotes its polyubiquitination

**Figure 2:**
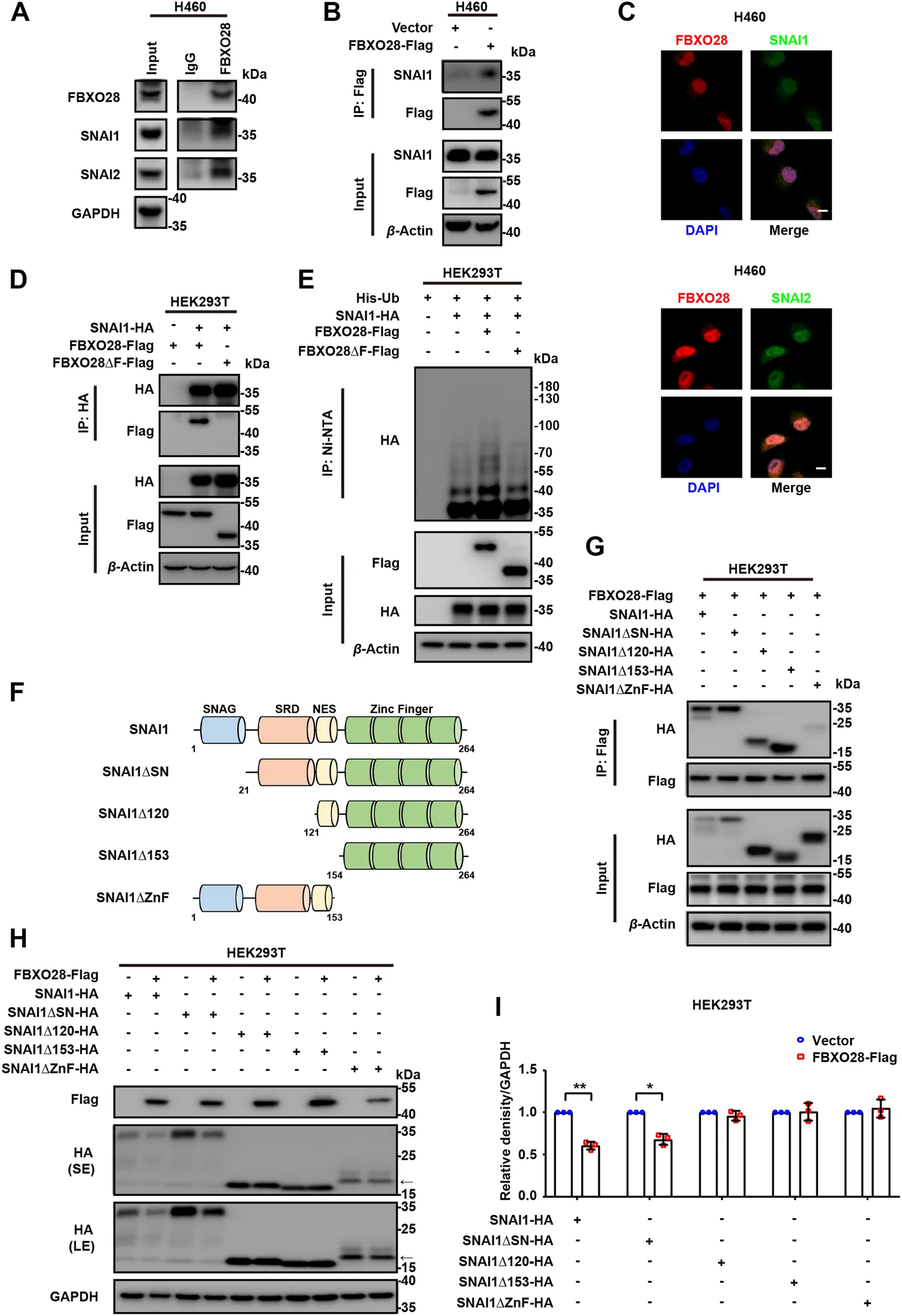
**FBXO28 interacts with and ubiquitinates SNAI1.***A*, H460 cells were subjected to IP with anti-FBXO28 antibody or IgG control, followed by IB analyses. *B*, H460 cells were transfected with FBXO28-Flag or control vector for 48 h prior to IP and IB analyses. *C*. Co-localization of FBXO28 (*red*), SNAI1/SNAI2 (*green*) by IF staining in H460 cells. Representative images are shown. Scale bar: 10 μm. *D*, IP and IB analyses in HEK293T cells co-transfected with indicated plasmids for 48 h. *E*. HEK293T cells were co-transfected with indicated plasmids for 72 h. The polyubiquitination proteins were purified by Ni-NTA beads and detected with anti-HA antibody. *F*, the schematic representation of SNAI1 deletion mutants. *G*. HEK293T cells were transfected with FBXO28-Flag and SNAI1 mutants for 48 h prior to IP and IB analyses. *H*, *I*. IB analyses of HEK293T cells transfected with indicated plasmids for 48 h. The arrows indicated target band. Representative images are shown in (*H*). SE: short exposure. LE: long exposure. The full-length or deletion mutants of SNAI2 protein levels were quantified and plotted in (*I*) as mean ± SD from three independent experiments. ∗*p* < 0.05 and ∗∗*p* < 0.01 (independent *t* test).

Next, we performed co-immunoprecipitation (co-IP) assays to examine whether FBXO28 interacts with SNAI1 in NSCLC cell line H460. Both endogenous and exogenously expressed FBXO28 efficiently associated with endogenous SNAI1, confirming the physical interaction between the two proteins (Fig. 2, *A* and *B*). Supporting our previous finding in HCC (22), endogenous or exogenous binding of FBXO28 with SNAI2 was also confirmed in H460 cells (Figs. 2*A* and S1*C*). We further performed IF analysis and observed that FBXO28 primarily co-localized with SNAI1 or SNAI2 proteins in the nucleus of the NSCLC cells (Figs. 2*A* and S1*D*).

Given that the F-box domain is essential for FBXO28-mediated recognition and ubiquitination of SNAI2 (22), we also assessed whether this domain is required for SNAI1 binding. Expectedly, deletion of the F-box domain in FBXO28 (FBXO28ΔF) markedly reduced its interaction with SNAI1 (Fig. 2*D*), indicating that the F-box domain is critical for SNAI1 and SNAI2 recognition. We next evaluated whether FBXO28 promotes SNAI1 polyubiquitination. Ubiquitination assays in HEK293T cells showed that overexpression of wild-type FBXO28 significantly enhanced SNAI1 polyubiquitination, whereas FBXO28ΔF failed to do so (Fig. 2*E*), demonstrating that FBXO28 facilitates SNAI1 polyubiquitination in an F-box-dependent manner.

To define the structural determinants within SNAI1 responsible for its interaction with FBXO28, we generated a series of SNAI1 deletion mutants including deletion of the SNAG domain (SNAI1ΔSN), co-deletion of the SNAG and serine-rich domains (SRD) (SNAI1Δ120), the C-terminal zinc finger domain (SNAI1Δ153) and deletion of thec (SNAI1ΔZnF) (Fig. 2*F*). Co-IP assays revealed that SNAI1ΔZnF failed to interact with FBXO28 (Fig. 2*G*), indicating that the zinc finger domain is critical for the binding between the two proteins.

Next, by analyzing the effect of FBXO28 on the protein levels of wild-type and mutant SNAI1, we observed that both the full-length SNAI1 and SNAI1ΔSN levels were markedly reduced, whereas the SNAI1Δ120, SNAI1Δ153, and SNAI1ΔZnF levels remained unaffected upon FBXO28 overexpression (Fig. 2, *H* and *I*). These data have suggested that, despite the SNAI1 lacking the zinc finger domain exhibiting resistance to proteolysis by FBXO28 as expected, there still exist critical residues within the 1 to 153 amino acid region of SNAI1 required for FBXO28-regulated stability.

### GSK3*β* is required for FBXO28-mediated degradation of SNAI1

**Figure 3:**
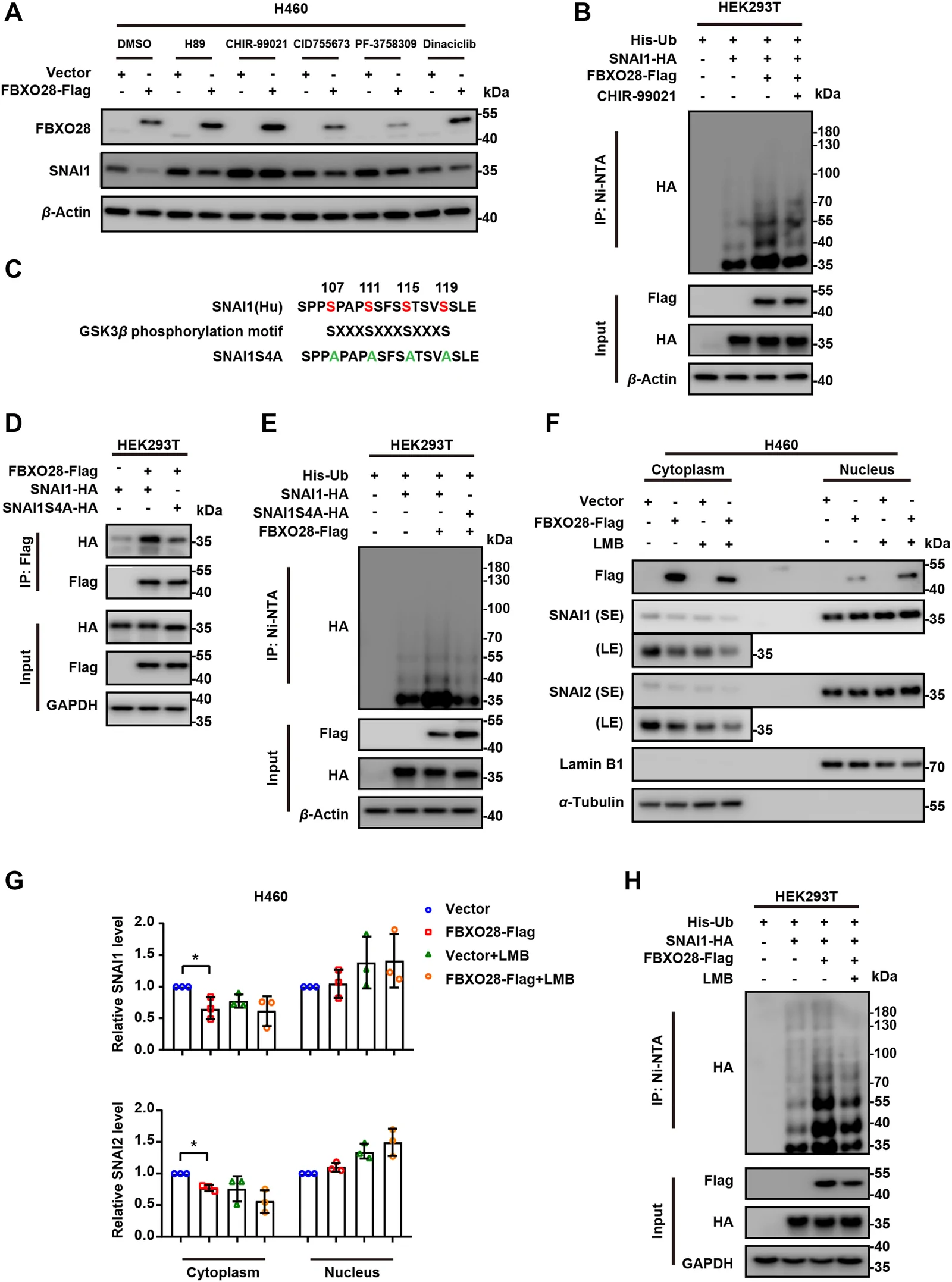
**FBXO28 promotes the ubiquitination of SNAI1 in cytoplasm, which is dependent on GSK3*β*-mediated phosphorylation.***A*, H460 cells were transfected with FBXO28-Flag or vector control for 24 h, followed by treatment with DMSO or 0.2 μM H89, 4 μM CHIR-99021, 5 μM CID755673, 0.5 μM PF-3758309, 2 nM Dinaciclib respectively for another 24 h before IB analyses. *B*, HEK293T cells were transfected with indicated plasmids for 48 h, followed by treatment with DMSO or 4 μM CHIR-99021 for another 24 h. The polyubiquitinated proteins were purified by Ni-NTA beads and detected with anti-HA antibody. *C*, a schematic representation of SNAI1S4A mutant. Phosphorylation sites by GSK3*β* were marked in *red*. *D*, IP and IB analyses in HEK293T cells co-transfected with indicated plasmids for 48 h. *E*, HEK293T cells were transfected with indicated plasmids for 72 h followed by purification with Ni-NTA beads and IB with anti-HA antibody. *F*, *G*, H460 cells were transfected with indicated plasmids for 48 h and treated with DMSO or LMB (100 ng/ml) for another 4 h. The cytoplasmic and nucleic fractions were isolated and subjected to IB analyses. SE: short exposure. LE: long exposure. The protein levels after normalized to *α*-Tubulin and Lamin B1 were quantified in (*G*) respectively. Data were expressed as mean ± SD from three independent experiments. ∗*p* < 0.05 (independent *t* test). *H*. HEK293T cells were transfected with indicated plasmids for 72 h prior to treatment with DMSO or LMB (100 ng/ml) for another 4 h, followed by purification with Ni-NTA beads and IB with anti-HA antibody.

Given that F-box proteins normally recognize phosphorylated substrates for degradation (23), we then reasoned that phosphorylation at the N-terminal region of SNAI1 may be critical for FBXO28-mediated decay. H460 cells were treated with a panel of candidate kinase inhibitors including CHIR-99021, CID755673, H89, PF-3758309, and Dinaciclib for GSK3*β*, PKD1, PKA, PAK, and CDK2, respectively (7, 8, 24, 25, 26). As seen in Figure 3*A*, only the GSK3*β* inhibitor CHIR-99021 effectively prevented the FBXO28-induced reduction in SNAI1 protein levels. Consistently, GSK3*β* inhibition markedly suppressed FBXO28-mediated polyubiquitination of the substrate in HEK293T cells (Fig. 3*B*). The findings indicate that GSK3*β*-dependent phosphorylation of SNAI1 is a prerequisite for FBXO28-mediated ubiquitination and degradation.

Previous studies have shown that GSK3*β* phosphorylates SNAI1 at four conserved serine residues (Ser107, Ser111, Ser115 and Ser119) (7, 10), which are located within the first 153 amino acids region (Fig. 3*C*). By generating a SNAI1 mutant in which the four serine residues were substituted with alanine (SNAI1S4A), we tested whether the residues are critical for FBXO28-dependent SNAI1 degradation. The expression of SNAI1S4A in HEK293T cells displayed markedly reduced binding affinity with FBXO28 when compared to the wild-type SNAI1 (Fig. 3*D*). Consistently, no detectable increase in the polyubiquitination of the SNAI1S4A was observed upon FBXO28 overexpression (Fig. 3*E*), supporting that FBXO28-mediated ubiquitination and degradation of SNAI1 depend on GSK3*β*-mediated phosphorylation. This also explains why SNAI1 deletion mutants, including SNAI1Δ120 and SNAI1Δ153, are resistant to FBXO28-induced degradation (Fig. 2, *H* and I).

Phosphorylation of SNAI1 at the four serine residues has been reported to generate a nuclear export signal, thereby promoting SNAI1 translocation from the nucleus to the cytoplasm (7). We next determined whether FBXO28 degrades the substrate at cytoplasm. The subcellular fractions of H460 cells overexpressed with FBXO28 were separated after treatment with the nucleus export inhibitor leptomycin B (LMB) for IB analysis. Indeed, a marked reduction in the cytoplasmic SNAI1 level was observed by upregulated FBXO28. Instead, LMB treatment primarily trapped more SNAI1 levels in the nucleus fraction, where FBXO28 exhibited negligible effects on the SNAI1 level (Fig. 3, *F* and G). Consistently, *in vivo* ubiquitination assays demonstrated that LMB treatment abolished FBXO28-induced polyubiquitination of SNAI1 (Fig. 3*H*). We similarly observed that the level of SNAI2 in the cytoplasmic context was greatly reduced rather than that in the nucleus fraction upon FBXO28 overexpression (Fig. 3, *F* and *G*). Collectively, these data support that GSK3*β*-mediated phosphorylation is required for FBXO28-mediated proteolysis of SNAI1, and that both SNAI1 and SNAI2 are degraded mainly at cytoplasm.

### FBXO28 controls the SNAI1 and SNAI2 turnover to suppress the migration and metastasis of NSCLC cells *in vitro* and *in vivo*

**Figure 4:**
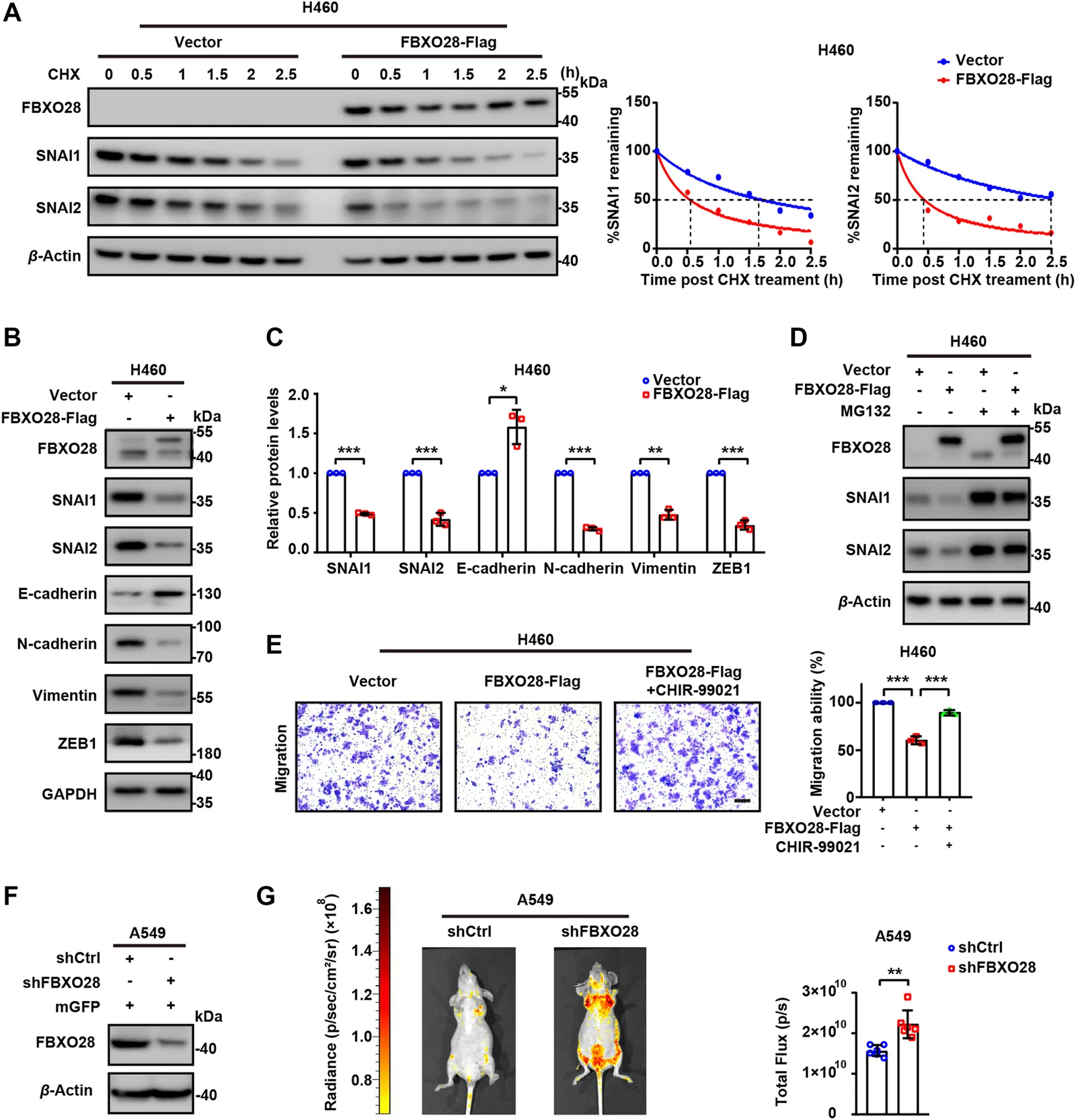
**FBXO28 negatively regulates SNAI1, SNAI2 protein levels and inhibits EMT pathway in NSCLC.***A*, H460 cells were transfected with indicated plasmids for 48 h followed by treatment with CHX (10 μg/ml) for indicated time interval before IB analyses. The SNAI1 and SNAI2 protein half-lives are shown in the *right panel*. *B*, *C*, H460 cells transfected with FBXO28-Flag or vector control for 48 h were subjected to IB analyses for detection of the EMT markers. The protein levels after normalized to GAPDH were quantified in (*C*) respectively. Data were expressed as mean ± SD from three independent experiments. ∗*p* < 0.05, ∗∗*p* < 0.01, ∗∗∗*p* < 0.001 (independent *t* test). *D*, H460 cells expressing FBXO28-Flag or vector control for 48 h were treated with MG132 (20 μM) for 6 h prior to IB analyses. *E*, H460 cells transiently expressing FBXO28-Flag or vector for 24 h were treated with DMSO or 4 μM CHIR-99021 for 24 h prior to migration assay. Data were expressed as mean ± SD from three independent experiments in the *right panel*. Scale bar: 10 μm. ∗∗∗*p* < 0.001 (one-way ANOVA). *F*, A549 stably expressing indicated vector for 72 h were subjected to IB analyses. *G*. A549 cells (2 × 10^5^) as in (*F*) were injected into BALB/c nude mice *via* the left cardiac ventricle, and systemic metastasis analysis was performed at the eighth week after injection (n = 6 per group). ∗∗*p* < 0.01 (independent *t* test).

Next, we assessed the effects of FBXO28 on the protein half-life of SNAI1 in lung cancer. FBXO28 overexpression or knockdown significantly shortened or prolonged the half-life of SNAI1 in NSCLC cells by CHX chase assays (Figs. 4*A* and S2*A*). Supportively, overexpression of SNAI1S4A demonstrated resistance to FBXO28-mediated decay relative to the wild-type SNAI1, as evidenced by its extended protein half-life in H460 cells (Fig. S2*B*). We also confirmed that FBXO28 overexpression shortened SNAI2 half-life in H460 cells (Fig. 4*A*). The FBXO28-mediated reduction in protein levels of both substrates was rescued by the proteasome inhibitor MG132, further supporting that degradation happened *via* the UPS (Fig. 4*D*).

By analyzing the protein levels of EMT markers, a marked reduction in the levels of mesenchymal markers N-cadherin, Vimentin and ZEB1 and an induction in the level of epithelial marker E-cadherin were revealed in FBXO28-overexpressing H460 cells (Fig. 4, *B* and *C*). Conversely, FBXO28 knockdown in H1299 cells exhibited opposite effects (Fig. S3, *A* and *B*). Also, the transcriptional levels of *CDH2* and *VIM* were significantly increased upon FBXO28 knockdown in H1299 cells, with the mRNA levels of *SNAI1* and *SNAI2* remaining unaltered (Fig. S3*C*). These data support that FBXO28 negatively regulates the level and function of SNAI1 and SNAI2 post-transcriptionally. Functionally, FBXO28 knockdown markedly enhanced migration, whereas FBXO28 overexpression suppressed the migratory capacity in the NSCLC cells as measured by the transwell assays (Fig. S3, *D–G*). Notably, co-treatment with CHIR-99021 greatly abrogated the inhibitory effect of FBXO28 on the migratory capacity in H460 cells (Fig. 4*E*), supporting the GSK3*β*-dependent decay of SNAI1. We further injected the mGFP-labeled A549 cells stably expressing shFBXO28 or a control vector into the left cardiac ventricle of BALB/c nude mice and performed systemic metastasis analysis at the eighth week post-injection. As shown in Figures 4, *F*, *G* and S3*H*, depletion of FBXO28 greatly enhanced the metastatic effect of A549 cells *in vivo*.

**Figure 5:**
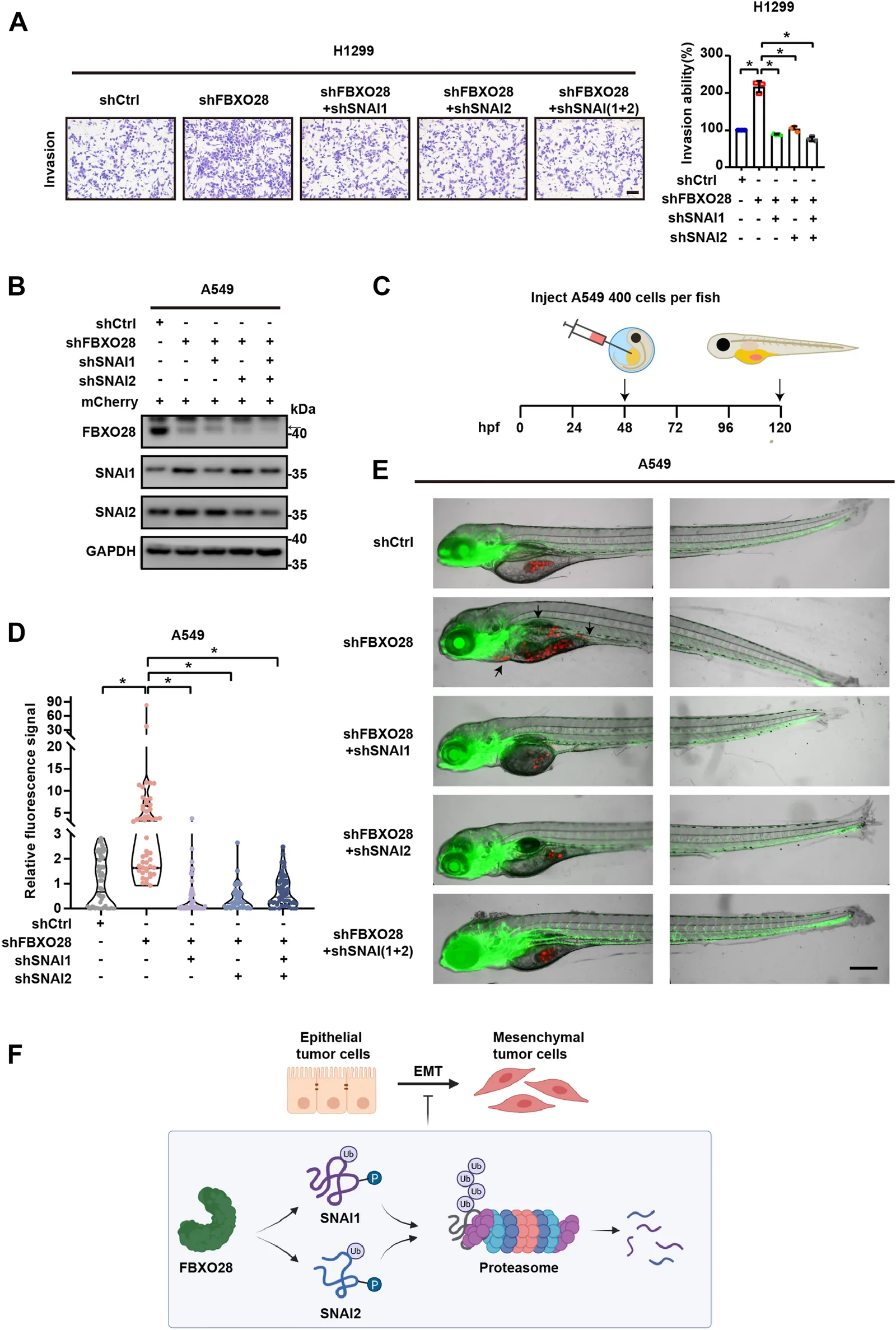
**Depletion of SNAI1 and SNAI2 reverses the enhanced metastasis capability caused by FBXO28 depletion in NSCLC.***A*, Transwell assay for the invasion ability of H1299 cells stably expressing shCtrl, shFBXO28 alone or in combination with shSNAI1 or/and shSNAI2. Representative images are shown (scale bar: 10 μm). Data were expressed as mean ± SD from three independent experiments. *∗p* < 0.05 (one-way ANOVA). *B*, A549 transduced with indicated lentivirus for 72 h were subjected to IB analyses. The arrow indicates target band. *C*, the schematic diagram illustrates the workflow for the zebrafish [*Tg* (*fli-1*:EGFP)] xenograft model using mCherry-labeled A549 cells as in (*B*). *D*, *E*, quantification of the fluorescence signal for the cell invasion after normalized to shCtrl (*D*) and representative images (*E*) of the different groups of zebrafish (n = 50) are shown. Arrows indicated cell mass dissemination or invasion. Scale bars: 250 μm. ∗*p* < 0.05 (one-way ANOVA). *F*, the working model. FBXO28 suppresses EMT and metastasis in cancer through targeting SNAI1 and SNAI2 for degradation *via* UPS (Created in BioRender. Wang, Z. (2026) https://BioRender.com/hieolwd).

Next, to determine whether SNAI1 and/or SNAI2 mediates the suppressive effects of FBXO28 on the invasion and metastasis of NSCLC cells, we generated H1299 cells stably expressing shFBXO28 alone or in combination with shSNAI1 and/or shSNAI2 *via* lentiviral transduction (Fig. S3*I*). Results of the transwell assays demonstrated that either depletion of SNAI1 or SNAI2 alone or co-depletion of both substrates greatly abrogated the enhanced invasion ability upon FBXO28 knockdown in H1299 cells (Fig. 5*A*). Moreover, the mCherry-labeled A549 cells expressing shFBXO28 alone or in combination with shSNAI1 and/or shSNAI2 were injected into the yolk sac of 48 hpf zebrafish embryos to establish xenografts in transgenic fluorescent zebrafish [*Tg (fli-1*:*EGF*P)] model, and fluorescence imaging of the zebrafish larva was captured at 120 hpf (Fig. 5, *B* and C). By examining the invasive and metastatic capability of A549 cells, we similarly found that knockdown of either substrate alone or both effectively reversed the enhanced invasion and metastasis induced by FBXO28 deficiency (Fig. 5, *D* and E). Collectively, these results demonstrate that FBXO28 controls the SNAI1 and SNAI2 turnover to suppress the migration and metastasis of NSCLC cells *in vitro* and *in vivo* (as seen in the working model in Fig. 5*F*).

## Discussion

In this study, we identify FBXO28 as a previously unrecognized E3 ubiquitin ligase that regulates SNAI1 stability and suppresses EMT-driven metastasis in NSCLC. We demonstrate that FBXO28 expression is reduced in cancer tissues, which is associated with a better prognosis of patients with NSCLC. Mechanistically, FBXO28 directly binds SNAI1 *via* its F-box domain, shortens the half-life of SNAI1 without affecting its transcription, and promotes its degradation. Importantly, this process requires prior GSK3*β*-mediated phosphorylation of SNAI1 and occurs selectively in the cytoplasmic compartment. These findings establish SNAI1 as an unidentified substrate of FBXO28 and reveal a new mechanism by which FBXO28 restrains NSCLC cell migration, invasion, and metastasis.

As intrinsically unstable proteins, the abundance of both SNAI1 and SNAI2 is tightly controlled by the UPS (27, 28, 29). To date, various F-box proteins, along with other types of E3 ubiquitin ligases (such as PPIL2, TRIM21, SPSB3, and DTX3L) have been reported to regulate SNAI1 stability in a variety of cancer types (7, 9, 10, 11, 12, 30, 31, 32, 33, 34, 35, 36). Moreover, a subset of E3 ubiquitin ligases including FBXO11, FBXO45, FBXW1, FBXL14, and TRIM21 are known to promote SNAI2 decay (11, 30, 37, 38, 39). Our work has shown that FBXO28 promotes SNAI2 degradation in HCC (22); a similar effect was observed in lung cancer. Importantly, we find SNAI1 to be another substrate for FBXO28-mediated degradation. Thus, our findings reveal FBXO28 as an unidentified E3 ligase that regulates the stability of both SNAI1 and SNAI2 (Fig. 5*F*), thereby expanding the regulatory landscape governing the proteins’ turnover in these malignancies. Considering that the E3 ubiquitin ligases known to regulate SNAI1 and/or SNAI2 stability in cancer are highly diversified, whether they function in a collaborative or competitive manner, or in a cell context-dependent way, remains unclear.

In the present work, we prove that FBXO28-mediated SNAI1 degradation requires GSK3*β*-dependent phosphorylation. Given that GSK3*β*-dependent phosphorylation normally promotes SNAI1 nuclear export (7), our findings confirm that FBXO28 selectively targets the cytoplasmic pool of SNAI1 for degradation. Interestingly, we also observed that SNAI2 was degraded by FBXO28 in the cytosol, suggesting that the possibility of PKA-mediated nucleus export for SNAI2 may exist as well. Notably, we found that depletion of either substrate is capable of fully reducing the increased invasion and metastasis effects by FBXO28 deficiency in NSCLC cells to a level comparable with the control group. Given that SNAI1 and SNAI2 are known to share conserved structural features and overlapping functions (27, 40), these results support that the two EMT regulators with redundant functions are independently rather than cooperatively regulated by FBXO28 in NSCLC. Similarly as our findings, either SNAI1 or SNAI2 knockdown fully abrogated the Pellino-1-mediated migration capacity in lung cancer cells (41).

In conclusion, this study uncovers a novel role of FBXO28 in controlling SNAI1 stability in NSCLC cells. These findings broaden the understanding of the role of FBXO28 and highlight this pathway as a potential therapeutic target for constraining metastasis in cancer.

## Experimental procedures

### Cell lines

H460, 1299, HEK293T and A549 cell lines were purchased from the National Infrastructure of Cell Line Resources in China. All cell lines were maintained in RPMI 1640 (for H460 and H1299), DMEM (for HEK293T) or Ham’s F12 (for A549) medium with 10% fetal bovine serum (FBS) and incubated at 37 °C in 5% CO_2_ incubator. Cells were authenticated by STR profiling regularly every half-year.

### Antibodies and reagents

Antibodies used in immunoblotting (IB) and immunoprecipitation (IP) assays are listed as below. anti-SNAI1 (#3879), anti-SNAI2 (#9585), anti-Vimentin (#5741), anti-E-cadherin (#3195), anti-N-cadherin (#13116), anti-ZEB1 (#3396), anti-HA (#3724), anti-DYKDDDDK (Flag) (#14793), Rabbit Anti-Mouse IgG (Light-Chain Specific) (#58802), Mouse Anti-Rabbit IgG (Light-Chain Specific) (#93702) and Mouse Anti-Rabbit IgG (Conformation Specific) (#5127) antibodies were purchased from Cell Signaling Technology. Anti-FBXO28 (#ab154068) and anti-Lamin B1 (#ab133741) antibodies were purchased from Abcam. Anti-GAPDH (#60004-1-Ig) and anti-*β*-Actin (#66009-1-Ig) antibodies were purchased from Proteintech. Anti-IgG (#sc2027) were purchased from Santa Cruz. Anti-*α*-Tubulin (#F0063) antibody, anti-HA magnetic beads (#B26202), anti-FLAG magnetic beads (#B26102), and Protein A/G magnetic beads (#B23202) were purchased from Selleck. For immunofluorescence (IF), anti-SNAI1 (#sc-2711977), and anti-SNAI2 (#sc-166476) antibodies were purchased from Santa Cruz. The Alexa Fluor 488-labeled Goat Anti-Mouse IgG (H + L) (#ZF-0512), Alexa Fluor 594-labeled Goat Anti-Rabbit IgG (H + L) (#ZF-0516), and DAPI (#ZLI-9557) were obtained from ZSGB-BIO.

Cycloheximide (CHX) (#S7418), MG132 (#S2619), Protein kinase A (PKA) inhibitor H89 (#S1582), Glycogen synthase kinase-3*β* (GSK3*β*) inhibitor CHIR-99021 (#S1263), Protein kinase D1 (PKD1) inhibitor CID755673 (#S7188), p21-activated kinase 1 (PAK1) inhibitor PF-3758309 (#S7094) and the cyclin-dependent kinase 2 (CDK2) inhibitor Dinaciclib (#S2768) were purchased from Selleck. Puromycin (#HY-B1743A) was purchased from MedChemExpress. Leptomycin B (LMB) (#50503ES08) was purchased from Yeasen.

### Plasmids and siRNAs

pCMV6-FBXO28-Myc-Flag (#RC206490), pLenti-C-mGFP-P2A (#PS100093), pRS-shSNAI1 (#TR309226A), pRS-shSNAI2 (#TR309225B) and pRS-shRNA control (#TR30012) plasmids were acquired from Origene. YOE-LV-mCherry-Control lentivirus was obtained from Ubigene Biosciences. pcDNA3.1-SNAI1-HA (#31697) was obtained from Addgene. FBXO28 and SNAI1 deletion and point mutants were obtained from Sangon. pLKO.1-shFBXO28 plasmid was constructed in our lab. The siRNA duplexes were synthesized by RiboBio. The sequences of siFBXO28, shFBXO28, shSNAI1 and shSNAI2 were as follows: siFBXO28: 5′- GACAAGAGGTTACCAAACT-3′, shFBXO28: 5′-TTATGTCCTACGACGAAATTA-3′, shSNAI1: 5′- TCTACTTCAGTCTCTTCCTTGGAGGCCGA-3′, shSNAI2: 5′- GAGGAATCTGGCTGCTGTGTAGCACACTG-3′.

### Cell transfection and transduction

For transient transfection, the plasmids or siRNAs were transfected with Neofect™ DNA transfection reagent (Neofect, #TF20121201) or riboFECT CP transfection kit (RiboBio, #C10511-05), respectively. For cell transduction, lentivirus vectors were co-transfected into HEK293T cells with lentivirus packaging plasmids (psPAX2 and pMD2.G) to produce lentivirus for stable knockdown of FBXO28, SNAI1, and SNAI2. The supernatants containing lentivirus were collected at 72 h after transfection. The transduced cells were selected with 5 μg/ml puromycin and maintained at 2 μg/ml puromycin. In addition, A549 cells expressing mGFP or mCherry tags were transduced with pLenti-C-mGFP-P2A or YOE-LV-mCherry-Control lentivirus, respectively, and stably selected.

### Migration and invasion assay

For migration assay, H460 and H1299 cells (3 × 10^4^ cells per chamber) in serum-free culture medium were seeded into the upper chambers of Transwell inserts (#3422, Corning). For invasion assay, H1299 cells (6 × 10^4^ cells per chamber) in serum-free medium were seeded into Matrigel-coated Transwell inserts (#354480, Corning). The medium containing 20% FBS was added to lower chambers. After 48 h of incubation, the chambers were fixed, stained and quantified as described previously (22).

### Tumor model in mice

For systemic metastasis analysis, six-week-old female BALB/c nude mice (GemPharmatech) were randomly assigned to two groups (n = 6 per group). Body weight was measured every 2 weeks. 2 × 10^5^ mGFP-labeled A549 cells were injected into the left cardiac ventricle. The bioluminescence imaging was analyzed at the eighth week post-injection using an IVIS Spectrum CT system (PerkinElmer), after which mice were sacrificed. Total flux [photons per second (p/s)] was quantified using Living Image software (version 4.4).

The animal experiment was approved by the Animal Experimentation Ethics Committee of the Institute of Medicinal Biotechnology, Chinese Academy of Medical Sciences, and conducted in strict compliance with the institutional regulations and operational procedures for experimental animal management.

### Tumor model in zebrafish

For the zebrafish [*Tg* (*fli-1*:EGFP)] xenograft models to assess the metastatic potential of NSCLC cells, approximately 400 mCherry-labeled A549 cells were microinjected into the perivitelline space of 48-h post-fertilization (48 hpf) zebrafish embryos. At 120 hpf, brightfield images along with fluorescent images of EGFP-labeled vasculature (green) and mCherry-labeled cancer cell masses (red) in the zebrafish larvae were acquired using a fluorescence microscope (DMI8, Leica). ImageJ was used to quantitatively analyze the intensity of red fluorescence. All measurements and analyses were conducted in a double-blinded manner to ensure objectivity.

### IB, IP, and IF assay

For IB and IP analysis, proteins were detected using indicated antibodies as previously described (42).For protein half-life analysis, cells were treated with 10 μg/ml cycloheximide (CHX) for the indicated time prior to IB analysis. For IF analysis, cells were seeded on coverslips and fixed with 4% paraformaldehyde. The proteins were stained with respective antibodies and cell nuclei were tained with DAPI. Images were captured using an Olympus FLUOVIEW FV4000 laser scanning confocal microscope with a 100 × oil immersion objective.

### Quantitative RT-PCR (qRT-PCR) analysis

qRT-PCR was conducted as described previously (43), with primer sequences in Table 1.

**Table 1:** Primers sequences of indicated genes

Gene	Forward sequence (5′-3′)	Reverse sequence (5′-3′)
*FBXO28*	TCCTCAGCTTTATGTCCTACGA	TGGGAGTTGTGCTTTAACTTGT
*VIM*	TGCCGTTGAAGCTGCTAACTA	CCAGAGGGAGTGAATCCAGATTA
*CDH2*	TGCGGTACAGTGTAACTGGG	GAAACCGGGCTATCTGCTCG
*SNAI1*	ACTGCAACAAGGAATACCTCAG	GCACTGGTACTTCTTGACATCTG
*SNAI2*	TGTGACAAGGAATATGTGAGCC	TGAGCCCTCAGATTTGACCTG
*GAPDH*	CATGAGAAGTATGACAACAGCCT	AGTCCTTCCACGATACCAAAGT

### Ubiquitination assay

The Ni-nitrilotriacetic acid (Ni-NTA) capture assay was conducted as described previously (22).

### Subcellular fractionation isolation assay

For the subcellular fractionation isolation assay, cells were transfected with the indicated plasmids for 48 h and subsequently treated with 100 ng/ml LMB for 4 h before harvesting. Nucleus and cytoplasmic fractions were isolated using the Nucleus and Cytoplasmic Protein Extraction Kit (#P0027, Beyotime, China) following the manufacturer's instructions, and subsequently analyzed by IB. Cytoplasmic and nucleic proteins were normalized to *α*-tubulin and Lamin B1, respectively.

### Human tissue analysis

Paired human NSCLC tissues were collected from Shanghai General Hospital (affiliated with Shanghai Jiao Tong University School of Medicine) with written informed consents obtained from all patients based on the Declaration of Helsinki. The study was approved by the Research Ethics Committee of Shanghai General Hospital and our Institute’s Ethics Committee before subjected to IB analysis.

### Bioinformatical analysis

For *FBXO28* expression analysis in cancer cell lines, *FBXO28* mRNA relative expression levels in epithelial and mesenchymal cancer cell lines were obtained from the Cancer Cell Line Encyclopedia (CCLE) database (44). Cell lines were classified into epithelial and mesenchymal subtypes based on established EMT signatures. Gene set enrichment analysis (GSEA) was performed using the clusterProfiler R package to compare the Kyoto Encyclopedia of Genes and Genomes (KEGG) pathways between *FBXO28*-high and *FBXO28*-low NSCLC cohorts (with mean cut-off).

### Statistical analysis

Quantitative data are presented as mean ± SD. Statistical comparisons between two groups were performed using a two-tailed Student’s *t* test, while multiple group comparisons were conducted using one-way ANOVA in SPSS (version 31.0). IB results shown were representative of at least three independent experiments. Statistical significance was evaluated at three levels: ∗*p* < 0.05, ∗∗*p* < 0.01, and ∗∗∗*p* < 0.001.

## Data availability

The Kaplan-Meier survival analyses were obtained from https://www.kmplot.com. TCGA data in Figures 1 *C*, *D* and S1, *A*, *B* were downloaded from http://xena.ucsc.edu/public/. All original immunoblots were provided as Supplementary material. All other raw datasets are available upon reasonable request.

## Supporting information

This article contains supporting information.

## Conflict of interest

The authors declare that they have no conflicts of interest with the contents of this article.
