# Supplementary material for: FBXO28 targets SNAI1 for ubiquitin-proteasomal degradation in non-small cell lung cancer
Source: J Biol Chem. 2026 Aug 13;302(10):113405. doi: 10.1016/j.jbc.2026.113405 (PMC13579309; doi:10.1016/j.jbc.2026.113405)
Supplement: Supplementary file 1 [file mmc1.docx]

**FBXO28 targets SNAI1 for ubiquitin-proteasomal degradation in non-small cell lung cancer**

**Jingyu Lin^1^**^#^**^△^, Xinran Qiao^1#^**^&^**, Hua Liu^2^**^#^**, Yang Chen^1^, Zhen Li^1^, Xinyi Tong, Mi Shen, Rui Zhang^1^, Ying Han^1^, Jiajia Shen^1*^, Zhen Wang^1*^**

1 Department of Biochemistry, Institute of Medicinal Biotechnology, Chinese Academy of Medical Sciences & Peking Union Medical College, Beijing, China.

2 Department of Thoracic Surgery, Shanghai General Hospital, Shanghai Jiao Tong University School of Medicine, No.100 Haining Road, Shanghai, China.

\# These authors contributed equally to this work.

**△**Current address: WuXi AppTec (Nantong) Co., Ltd., Nantong, China

&Current address: Beijing Tri-Prime Gene Pharmaceutical Co., Ltd., Beijing, China.

*Correspondence to: Jiajia Shen, No.1 Tian Tan Xi Li, Beijing 100050, China. Tel: 86-10-63162129, Email: shenjiajia@imb.pumc.edu.cn; Zhen Wang, No.1 Tian Tan Xi Li, Beijing 100050, China. Tel: 86-10-63165289, Email: [wangzhen@imb.pumc.edu.cn](mailto:wangzhen@imb.pumc.edu.cn).

**Supplementary Data**

**Figure S1. GSEA analysis for *FBXO28* expression in Lung Squamous Cell Carcinoma (LUSC) and interaction of FBXO28 with SNAI1 or SNAI2.**

A, B. GSEA analysis of TCGA datasets for LUSC. C. H460 cells were transfected with FBXO28-Flag or control vector for 48 h prior to IP and IB analyses. D. Co-localization of FBXO28 (red), SNAI1 or SNAI2 (green) by IF staining in H1299 cells. Representative images are shown. Scale bar: 10 μm.

**Figure S2. GSK3*β*-dependent phosphorylation is indispensable for FBXO28-mediated SNAI1 degradation.**

A. H1299 cells stably expressing shFBXO28 or shCtrl were treated with CHX (10 μg/mL) for the indicated time interval and subjected to IB analyses. The SNAI1 protein half-lives are quantified and shown in the right panel. B. H460 cells were transfected with indicated plasmids for 48 h followed by treatment with CHX for the indicated time interval before IB analyses. The protein levels were quantified and shown in the right panel.

**Figure S3. FBXO28 inhibits migration of NSCLC cells through negatively regulating SNAI1 protein levels.**

A, B. H1299 stably expressing shFBXO28 or shCtrl were subjected to IB analyses for detection of the EMT markers. The protein levels after normalized to GAPDH were quantified in (B) respectively. Data were expressed as mean ± SD from three independent experiments. **P* < 0.05, ***P* < 0.01, (independent *t*-test). C. qRT-PCR analyses of the EMT markers in H1299 cells stably expressing shFBXO28 or shCtrl. Data were presented as mean ± SD of three independent experiments, **P* < 0.05 and ****P* < 0.001 (independent *t*-test). D. IB analyses of FBXO28 levels in H1299 cells transfected with siFBXO28 or siCtrl for 48 h. E. Migration assay for H1299 cells as in (D). Representative images are shown (scale bar: 10μm). Data were expressed as mean ± SD of three independent experiments. **P* < 0.05 (independent *t*-test). F. IB analyses of FBXO28 levels in H460 cells transfected with FBXO28-Flag or vector for 48 h. G. Migration assay for H460 cells as in (F). Representative images are shown (scale bar: 10μm). Data were expressed as mean ± SD of three independent experiments. **P* < 0.05 (independent *t*-test). H. The body weight of mice was measured every two weeks during the indicated time and plotted (n = 6 per group). I. H1299 cells stably expressing shCtrl, shFBXO28 alone or in combination with shSNAI1 or/and shSNAI2 were subjected to IB analyses.
